# The Effects of Platelet-Rich and Platelet-Poor Plasma on Biological Characteristics of BM-MSCs In Vitro

**DOI:** 10.1155/2020/8546231

**Published:** 2020-08-26

**Authors:** Jiahui Zhang, Jun Zhang, Nannan Zhang, Tao Li, Xiaohe Zhou, Jue Jia, Yingying Liang, Xiaochun Sun, Huabiao Chen

**Affiliations:** ^1^School of Medicine, Jiangsu University, Zhenjiang, Jiangsu 212001, China; ^2^Department of Laboratory, The Affiliated Hospital of Yangzhou University, Yangzhou, Jiangsu 225001, China; ^3^The Affiliated Hospital of Jiangsu University, Zhenjiang, Jiangsu 212001, China; ^4^Vaccine and Immunotherapy Center, Experimental Therapeutics and Molecular Imaging Laboratory, Massachusetts General Hospital, Harvard Medical School, Boston, MA 02114, USA

## Abstract

Platelet-rich plasma (PRP) and its byproduct platelet-poor plasma (PPP) are rich sources of cytokines in tissue damage repair. Bone marrow-derived mesenchymal stem cells (BM-MSCs) have received more and more attention for their ability to treat multiple diseases. The purpose of our study was to investigate the biologic action of PPP and PRP on BM-MSCs. The adipogenic potential of BM-MSCs revealed no obvious change, but the osteogenic ability of BM-MSCs was enhanced after treated with PRP. CCK8 assays and cell colony formation assays showed that PRP promoted cell proliferation, while this effect of PPP was not obvious. No obvious difference was found in cell cycle and apoptosis of BM-MSCs between PRP and PPP treatment. Expression of *β*-galactosidase, a biological marker of senescence, was decreased upon PRP treatment which indicated that PRP provided significant protection against cellular senescence. The migratory capacity of BM-MSCs was detected by scratch and transwell assays. The results indicated that PRP could affect the migration ability of BM-MSCs. From immunofluorescence detection and western blot, we demonstrated that the level of epithelial-mesenchymal transition-related proteins was changed and several pluripotency marker genes, including Sox2, Sall4, Oct4, and Nanog, were increased. Finally, the expression of the key signal pathway such as PI3K/AKT was examined. Our findings suggested that PRP promoted cell migration of BM-MSCs via stimulating the signaling pathway of PI3K/AKT.

## 1. Introduction

Platelet-rich plasma (PRP) and platelet-poor plasma (PPP) are fractions of blood plasma with different platelet concentrations. The platelet content of PRP and PPP is 6 × 10^11^ platelets/ml and 0.5 × 10^8^ platelets/ml, respectively. PPP and PRP are obtained by repeatedly centrifuging and washing whole blood of humans at different centrifugal speeds. They are prepared by mixing different concentrations of platelets and anticoagulants. There are many methods to extract PPP and PRP, such as secondary centrifugation, PCCS kit, and Curasa methods. PRP contains a lot of cytokines, for instance, EGF, TGF-*β*, PDGF, and IGF-1. These cytokines play a significant role in supporting and stimulating growth and differentiation of mesenchymal stem cells [1]. Studies have shown that PRP has cell adhesion molecules and chemotactic properties, which recruit MSCs to the repair site of musculoskeletal injury [2]. PRP has been used for regenerative injection therapy of osteoarthritis causing symptomatic relief in early osteoarthritis and improving clinical outcome [3]. Clinically, PRP in conjunction with MSCs is applied to partial, full, osteochondral, and osteoarthritic defects [4]. Of the numerous cytokines and growth factors present in PRP lysate, parts of them are conducive to maintain the phenotype of chondrocytes, others are not. Meanwhile, PRP is proved to have both pro- and anti-inflammatory effects [5]. A number of investigations have revealed that PRP could promote cell migration; maintain the adipogenic, chondrogenic, and osteogenic differentiation capacity of stem cells; enhance cell clone formation; and maintain an immunosuppressive state [6, 7].

PPP, as a centrifugation byproduct of anticoagulated blood, has lower platelet concentration than normal blood. The main components of PPP are fibrinogen, fibronectin, and thrombin. The biological effects of PPP are to participate in hemostasis and coagulation, to act as a cell attachment vector, and also to promote mitosis of fibroblasts and epithelial cells [8]. Although PPP is not as concentrated in platelets as PRP, it has been demonstrated that PPP can sustain cell growth and survival as well. PPP promotes wound healing-associated cell functions and accelerates cell migration and proliferation of fibroblasts [9–11]. Currently, many investigators have used PPP to culture BM-MSCs, HUC-MSCs, or ADSCs, instead of using serum from bovine or other sources. At the clinical level, platelet-related products including platelet-rich fibrin, PRP, and PPP are widely used to promote wound healing and tissue regeneration [12, 13]. Given the above research, it is important to further study the differences between PPP and PRP in terms of composition and biologic effects.

In recent years, the plasticity of BM-MSCs has received considerable attention. BM-MSCs have gained popularity for their potential as seed cells to treat various human diseases, including wound repair. MSCs could differentiate into many types of tissue cells and contribute to the regeneration of bone, muscle, and adipose tissues [14, 15]. In addition, many studies have shown that BM-MSCs release plentiful nerve growth factors, interleukin-10, and monocyte chemoattractant protein-1 to stimulate BM-MSCs' growth and survival. Due to the plasticity of BM-MSCs, the use of PRP containing BM-MSCs as a potential therapeutic strategy to induce formation of new bone tissue may have a synergistic regenerative effect and is an increasingly attractive area of research in regenerative medicine. Based on this knowledge, we decided to investigate the biological effects of PPP and PRP on BM-MSCs in vitro. In this study, we analyzed the potential of PPP and PRP in regulating the expansion, differentiation, migration, and protein expression of stem cells. To achieve this aim, morphological experiments were used to monitor changes in treatment groups and the relevant signaling pathways of cell migration were explored.

## 2. Materials and Methods

All Sprague Dawley rats used in our research were performed under approval of the University Committee on Use and Care of Animals of Jiangsu University (2014280).

### 2.1. Isolation and Characterization of BM-MSCs

Primary BM-MSCs were gained from the femoral cavity of 80-100 g male Sprague Dawley rats and cultured according to standard protocols [16]. The cells were rinsed twice with PBS and cultured in low-glucose Dulbecco's modified Eagle's medium (HyClone, China), 10% fetal bovine serum (Excell, Australia), and 100 U/ml penicillin and streptomycin (Shandong, China) at 37°C in a humid 5% CO_2_ air atmosphere. After 4 days, nonadherent cells were discarded by adding a new culture medium. When BM-MSCs reached 80% confluence, they were trypsinized with 0.25% trypsin (Biological, USA) and cultured for further expansion. All BM-MSCs used in this study were third generation. The phenotypes of BM-MSCs were tested by Attune NxT (Invitrogen, USA) after labeled with fluorochrome-conjugated antibodies, including CD29, CD44, CD90, CD105, CD34, CD45, CD133, and HLA-DR (Becton, USA). For evaluation of differentiation capacity, third-passage BM-MSCs were pretreated with adipogenic and osteogenic induction medium (Cyagen, China). The adipocytes were then stained with Oil Red O (Cyagen, China) and alkaline phosphatase (Shanghai, China) to assess adipogenic and osteogenic differentiation.

### 2.2. PPP and PRP Preparation

All PPP and PRP used in the experiments were provided by the central blood bank of Zhenjiang city, Jiangsu, China. Throughout the manuscript, we used PRP at a concentration of 100 × 10^6^ platelets/ml.

### 2.3. Cell Proliferation Assay

The cells were divided into three groups: the blank group, the control group, and the experimental group. The blank group added serum-free L-DMEM only, the control group added the same volume of plasma as the experimental group, and the experimental group received different concentrations of PRP (in platelets/ml, 5 × 10^6^/ml, 25 × 10^6^/ml, 50 × 10^6^/ml, 100 × 10^6^/ml, 200 × 10^6^/ml, and 400 × 10^6^/ml). CCK8 (Vazyme, China) assays were performed at 0 h, 24 h, 48 h, 72 h, and 96 h according to the manufacturer's instructions, with 3 × 10^3^ cells/well seeded in cell culture plates. CCK8 solution was added to 96-well plates and incubated for 4 h. The absorbances of each well were read at 450 nm using an enzyme-linked immunosorbent assay plate reader (BioTek, USA).

### 2.4. Cell Colony Formation Assay

Cells at a density of 1000 cells/cm^2^ were plated in cell culture plates and attached overnight at a cell incubator. The medium for cells in the experimental group was changed to serum-free L-DMEM supplemented with PRP at 100 × 10^6^ platelets/ml, while cells in the blank group were maintained in L-DMEM. Then, the culture medium was replaced with fresh medium after treated with PRP for 48 h and the medium was changed every 3 days for the next 2 weeks. The BM-MSCs were fixed for 30 min using 4% paraformaldehyde and stained for 20 min using crystal violet. The number of cell colonies was observed and counted under a microscope for statistical analysis.

### 2.5. Morphological Observation

At the third generation, BM-MSCs (2 × 10^5^ per well) were seeded in cell culture plates with 10% FBS/L-DMEM in a humid 5% CO_2_ air atmosphere. After attached overnight, the serum-free PRP (100 × 10^6^ platelets/ml) was added in 24-well plates for 48 h. The morphological changes of BM-MSCs were observed by an inverted microscope (Nikon, Japan) at 40x magnification.

### 2.6. Senescence-Associated *β*-Galactosidase Staining

Cell senescence was analyzed by an SA-*β*-gal staining kit (Beyotime, China) after coculture with serum-free PRP (100 × 10^6^ platelets/ml). Cells were fixed with 4% formaldehyde and washed with PBS. Afterwards, SA-*β*-gal stain solution was added to six-well plates and the plates were incubated in a CO_2_-free chamber. The positive cells were stained blue. The percentage of stained cells was counted from six fields.

### 2.7. Multidifferentiation Capacity In Vitro

#### 2.7.1. Adipogenic Differentiation Induction

BM-MSCs were plated in cell culture plates at a density of 2 × 10^4^ cells/cm^2^ and cultured in serum-free PRP (100 × 10^6^ platelets/ml) for 48 h. When cells reached 70% confluence, they were cultivated with a new medium containing the adipogenic induction medium (10 *μ*g/ml insulin, 0.5 mM IBMX, 200 *μ*M indomethacin, and 1 *μ*M dexamethasone) for 21 days. The formation of oil droplets was examined using Oil Red O (Cyagen, China) staining and imaged by an inverted microscope.

#### 2.7.2. Osteogenic Differentiation Induction

Cells at passage three were seeded in six-well plates at a density of 2 × 10^4^ cells/cm^2^. After incubation at 37°C with serum-free PRP (100 × 10^6^ platelets/ml), the medium was replaced with the osteogenic induction medium (0.1 *μ*M dexamethasone, 4 *μ*g/ml basic fibroblast growth factor, 10 mM *β*-glycerophosphate, and 50 *μ*g/ml ascorbic acid) and the medium was changed every 3 days. The cells were stained with Alizarin Red (Cyagen, China), and the stained calcium deposits in BM-MSCs appeared red under a light microscope.

### 2.8. Cell Cycle Assay

Cells were added in cell culture plates at 3 × 10^4^ cells/well and treated with serum-free PRP (100 × 10^6^ platelets/ml) for 48 h. The cells were then washed using cold PBS and stained with propidium iodide (Sigma, USA) for 30 min in the dark. The stained cells were detected by flow cytometry (Becton, USA).

### 2.9. Cell Apoptosis Assay

The Annexin V/FITC Apoptosis Detection Kit (Sigma, USA) was used to evaluate the effect of PPP and PRP on cell apoptosis. Briefly, after 48 h of culture with serum-free PRP (100 × 10^6^ platelets/ml), the cells were harvested and stained with PI and Annexin V-fluorescein isothiocyanate (FITC) for 20 min on ice in the dark. The apoptotic cells were analyzed individually by flow cytometry (Becton, USA).

### 2.10. Transwell Migration Assay

To test the function of PPP and PRP on the migration of BM-MSCs, we carried out transwell assays based on the manufacturer's instructions (Corning, USA) with slight modifications. Firstly, the cells (3 × 10^4^ per well) were suspended in the serum-free medium on the upper chamber of transwell membranes after pretreated with serum-free PRP (100 × 10^6^ platelets/ml). Next, the medium of 10% FBS was added to the lower chamber and the cells were cultivated for 16 h. Cells which remained on the upper surface of the filter were wiped off. Finally, the migrated cells on the lower surface were fixed for 30 min using paraformaldehyde and stained with 0.5% crystal violet for 5 min. The migrated cells were observed and counted under a microscope.

### 2.11. Scratch Assay

Cells pretreated with serum-free PRP (100 × 10^6^ platelets/ml) for 48 h were seeded in cell culture plates at a concentration of 1 × 10^5^ per well. When the cells reached 80% confluence, a P200 pipette was used to scratch off an area of BM-MSCs from the confluent growth. The scratched area had a fixed width. We used the serum-free medium to rinse the six-well flat-bottom plate and added 10% FBS L-DMEM to cultivate the cells. The scratch area was photographed at 0, 12, 24, and 36 h. The cell migration ratio was expressed as a percentage of wound closure: %of wound closure = [(*A*_*t*_ = 0 h − *A*_*t*_ = 36 h)/*A*_*t*_ = 0 h] × 100%, where *A*_*t*_ represents the width of the scratch wound.

### 2.12. Immunofluorescence

Immunofluorescence was carried out to determine the expression of proteins related to EMT. BM-MSCs were fixed for 10 min using paraformaldehyde and permeabilized with 0.1% Triton X-100 for 10 min, then soaked in 5% BSA, and incubated with rabbit anti-rat antibodies E-cadherin (1 : 150), N-cadherin (1 : 150), Vimentin (1 : 200), PI3K (1 : 200), AKT (1 : 200), GSK-3*β* (1 : 200), and *β*-catenin (1 : 200) (CST, USA) and goat anti-mouse *α*-SMA antibody (1 : 100) (CST, USA) at 4°C overnight. The BM-MSCs were washed and incubated with Alexa Fluor 555-conjugated donkey anti-rabbit IgG or FITC-conjugated goat anti-rabbit IgG (Invitrogen, USA) for 1 h. The nuclei were stained with DAPI (Sigma, USA), and images were obtained by a fluorescent microscope (Nikon, Japan).

### 2.13. Western Blot

Cells were lysed in RIPA buffer containing proteinase inhibitors. Equal amount of proteins was separated by a 12% SDS-PAGE gel. Following electrophoresis, proteins were transferred to a PVDF membrane, blocked in 5% nonfat milk, and incubated with primary antibodies at 4°C overnight [17]. The membranes were cultured with primary antibodies against rabbit anti-rat Sox2, Sall4, Oct4, Nanog (Bioworld Technology, diluted at 1 : 500), E-cadherin, N-cadherin, Vimentin, *α*-SMA, PI3K, AKT, GSK-3*β*, and *β*-catenin (CST, diluted at 1 : 800), respectively. The secondary antibodies were HRP-conjugated goat anti-rabbit and goat anti-mouse antibodies (ABclonal, 1 : 2000) at 37°C for 1 h after rinsing with TBST. Signals were developed using an enhanced Chemiluminescent Protein Detection Module.

### 2.14. Statistical Analysis

Our experiments were implemented in triplicate, which have been repeated for three times. All quantitative data are expressed as mean ± SD. The significance of the difference between groups was tested by GraphPad Prism 7.0 (GraphPad, USA). *P* < 0.05 was accounted statistically significant.

## 3. Results

### 3.1. Characterization of BM-MSCs and Preparation of PPP/PRP

**Figure 1:**
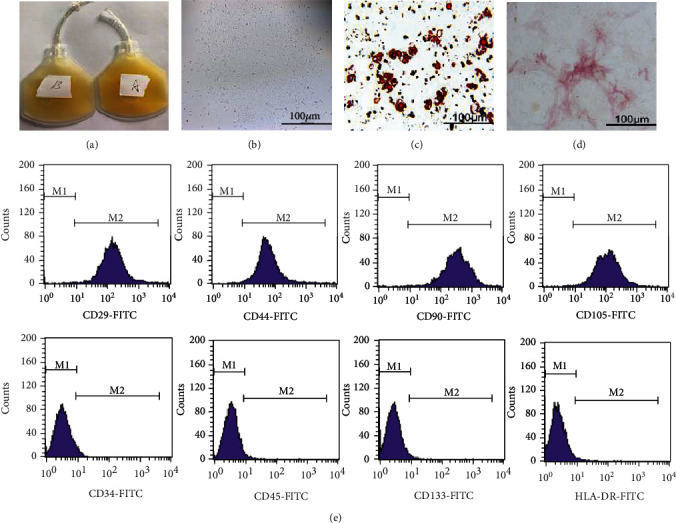


All PPP and PRP used in the experiments were provided by the central blood bank of Zhenjiang city, Jiangsu province (Figure 1(a)). After being obtained from the femur and cultured for 14 days, the BM-MSCs presented an elongated and fibroblast morphology (Figure 1(b)). The cytoplasm of BM-MSCs is transparent, and it was difficult to clearly observe the difference between cells by ordinary microscopes. Afterwards, BM-MSCs were cocultured with the adipogenic or osteogenic medium and stained with Oil Red O or ALP (Figures 1(c) and 1(d)). Flow cytometry results show that the expression of CD29, CD44, CD90, and CD105 was increased, while the expression of CD34, CD45, CD133, and HLA-DR was decreased (Figure 1(e)).

### 3.2. PRP Promotes Cell Proliferation and Protects against Cell Senescence

**Figure 2:**
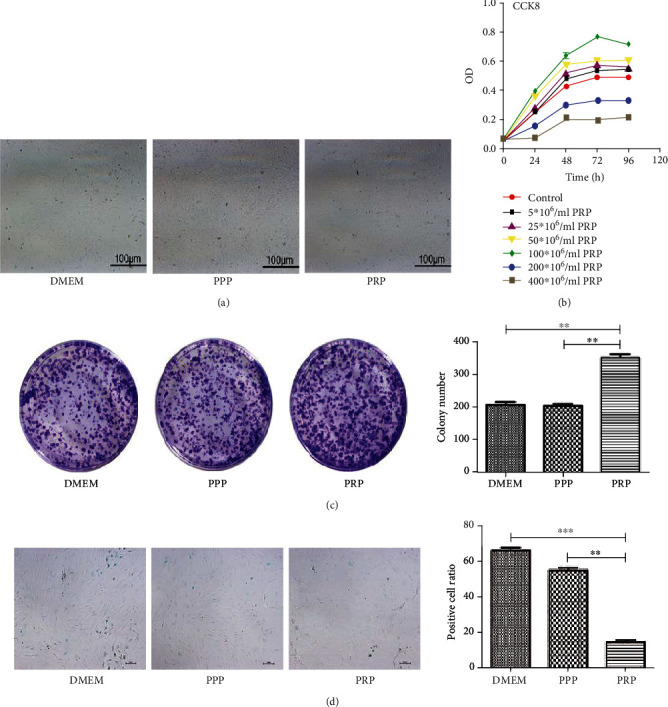


In the PRP group, we watched that the connections between BM-MSCs are getting tighter. However, these changes were not found in the DMEM group and the PPP group (Figure 2(a)). For the sake of investigating the effect of PPP and PRP on cell proliferation, we performed CCK8 and cell clone formation experiments. We discovered that PRP in suitable concentration and culture duration (100 × 10^6^ platelets/ml for 48 h) could promote the growth of BM-MSCs. These results demonstrated that PRP enhanced cell proliferation and cell cloning ability while PPP had no obvious effect (Figures 2(b) and 2(c)). Senescence-associated *β*-galactosidase staining was applied to measure the effects of PPP/PRP on BM-MSCs, and the results revealed that the DMEM group and PPP group had more senescent cells while the PRP group obviously exhibited less senescent cells, indicating that PRP protected BM-MSCs from cell senescence (Figure 2(d)). In the PRP group, BM-MSCs had stronger cloning ability and the cells were crowded, so the cell morphology became smaller.

### 3.3. PRP at a Suitable Concentration Could Accelerate Cell Migration

**Figure 3:**
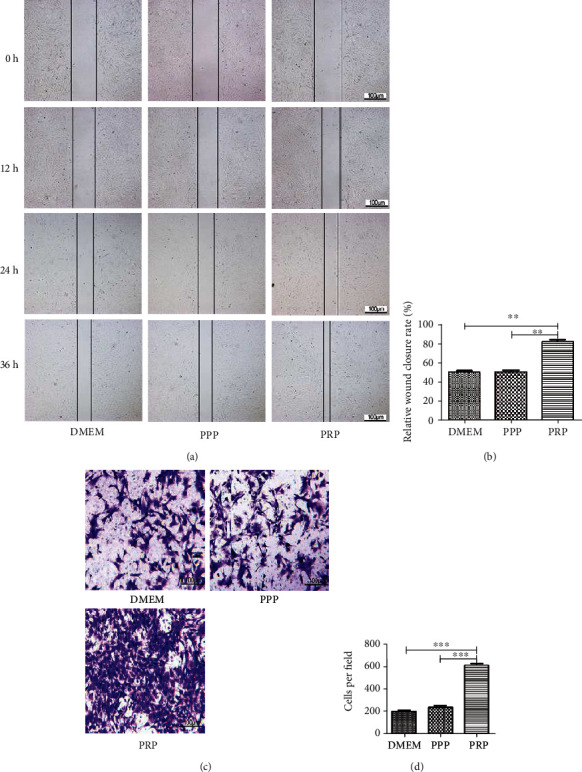


Firstly, we separated the cells by scratch wounds, and the rate of cell migration was observed at 36 h. From the results analyzed with the ImageJ program, we found that cell migration in the experimental group (PRP at 100 × 10^6^ platelets/ml) was enhanced compared to the DMEM group and the PPP group (Figures 3(a) and 3(b)). In transwell assays, we observed that migrating cells were dramatically increased after pretreated with PRP and statistical analysis displayed a remarkable difference between groups (Figures 3(c) and 3(d)).

### 3.4. The Effect of PPP and PRP on Multidifferentiation Capacity, Cell Cycle, and Apoptosis

**Figure 4:**
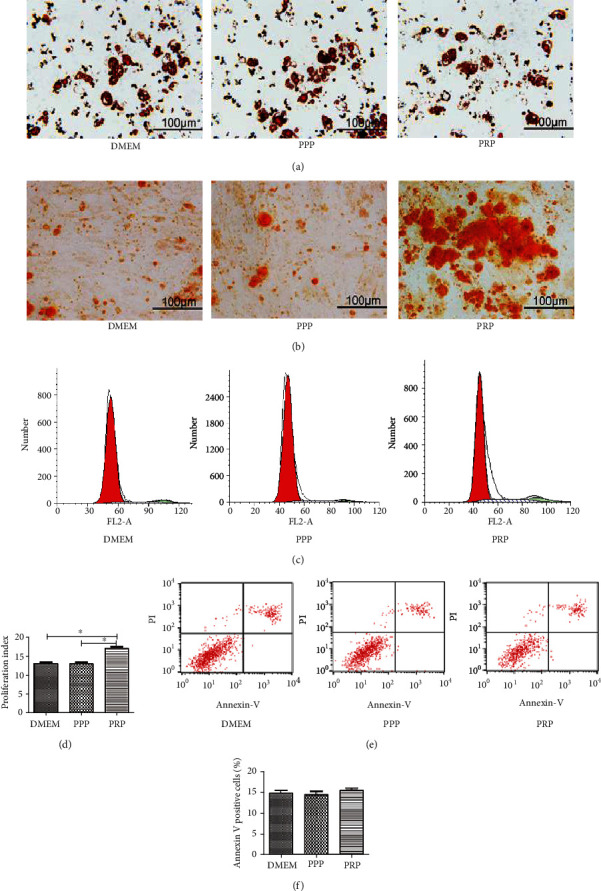


In vitro, the multidifferentiation capacity of BM-MSCs was assessed through induction of adipogenic and osteogenic differentiation. The ratio of stained cells was not significantly different among groups (Figure 4(a)), while the percentage of Alizarin Red-positive cells in the PRP group was more than that in the DMEM group or PPP group (Figure 4(b)). The effect of PPP and PRP in regulating the cell apoptosis and cycle was detected by flow cytometry. Cell cycle assays showed that the percentages of labeled cells in S or G2-M phase were higher than the control group (Figures 4(c) and 4(d)). From the cell apoptosis assay, we found that the percentage of apoptotic cells was similar between groups, and the histogram analysis also showed no statistically significant difference (Figures 4(e) and 4(f)). The above experiments confirmed that PRP can significantly promote cell osteogenic differentiation, while PRP at the density of 100 × 10^6^ platelets/ml had no obvious promotional or inhibitory effect on adipogenic differentiation, cell cycle, or apoptosis.

### 3.5. PRP Upregulates the Expression of Related Proteins and Promotes Cell Migration by Activating the PI3K/AKT Signaling Pathway

**Figure 5:**
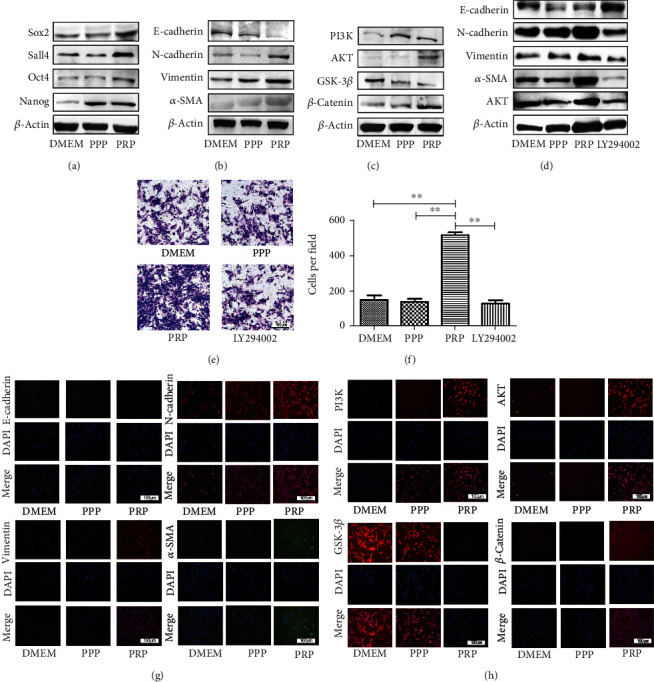


Western blot was applied to characterize the level of stem cell-related proteins in BM-MSCs. When measured at 48 h posttreatment, we discovered that PRP markedly increased the expression of Sox2, Sall4, Oct4, Nanog, N-cadherin, Vimentin, and *α*-SMA yet significantly decreased the level of E-cadherin (Figures 5(a) and 5(b)). Similarly, the result of western blots showed changes consistent with the transwell and scratch assays. A previous study has demonstrated that the PI3K/AKT signaling pathway plays a significant role in controlling cell proliferation, angiogenesis and cell migration [18]. Therefore, we suspected that it would be an important mechanism underlying PRP regulation of cell migration. The level of key molecule in the PI3K/AKT signaling pathway was examined as well. After PRP stimulation, we were surprised to discover that when PI3K was activated it significantly promoted the expression of AKT. AKT upregulation increased the expression of *β*-catenin by downregulating the expression of GSK-3*β* (Figure 5(c)). We then added the AKT pathway inhibitor LY294002 to block the activation of AKT. We found that LY294002 almost completely blocked the expression of E-cadherin, N-cadherin, Vimentin, *α*-SMA and AKT, while cell migration significantly decreased (Figure 5(d)–5(f)). In addition, the results of immunofluorescence detection revealed changing patterns similar to the western blot results (Figures 5(g) and 5(h)), suggesting that PRP possibly activates the PI3K/AKT signaling pathway in BM-MSCs.

## 4. Discussion

Lately, autologous blood-derived fractions, for instance, PRP, PPP, and PRF, have been widely applied to repair tissue damage and wound healing as a cytokine store [19–21]. As is well known, activated PRP includes a mass of cytokines, such as EGF, SRIH, and FGF [22, 23]. It has been reported that PRP could promote new bone growth by releasing PDGF and TGF-*β* [24]. PDGF is the first factor in bone healing, which can stimulate the proliferation of MSCs, increase the number of osteoblasts, and promote the secretion of extracellular matrix [25]. TGF-*β* could enhance the number and activity of osteoblasts transformed from MSCs [26]. BMP derived from PRP has a strong effect on the differentiation of MSCs, suggesting that BMP could induce the phenotype transformation of undifferentiated cells into osteoblasts [27]. MSCs obtained from the mesoderm are multipotent stem cells and important seed cells in tissue engineering [28]. MSCs could efficiently facilitate angiogenesis, ameliorate carbon tetrachloride-induced hepatic injury, and repair acute kidney injury [29, 30]. Furthermore, BMP-2-derived PRP combining with MSCs enhanced bone formation more effectively than did either treatment alone. Nao et al. speculated that gelatin *β*-TCP sponges incorporating BMP-2, MSCs, and PRP in a bilayered structure would provide optimal bone and cartilage regeneration and thus improve joint healing [31]. Actually, studies have reported that PRP can attract MSCs, and this phenomenon is called the chemotactic effect [32]. Another promising area of research also observed that PRP was able to raise other cells and promote the initial healing of wounds [33]. From the experiments, we discovered that they promoted cell osteogenic ability to different degrees but restrained cell adipogenic ability, which is consistent with other studies. A study explained that PRP could improve cell cycle and inhibit cell apoptosis [34]. However, in our study, we failed to find an obvious effect on the cell cycle and apoptosis after pretreated with PPP and PRP. We speculated that this failure might be due to the differences in PPP and PRP concentration between the different studies. Therefore, we verified that PRP attenuated cell senescence during the early phase of cell growth, as shown by the results of senescence-associated *β*-galactosidase staining. Reports with regard to the function of PRP have mainly focused on its promotional effects on cell growth. For instance, many studies have reported that PRP could facilitate cell migration and boost cell proliferation [35]. Other reports demonstrated that PRP was able to recruit MSCs to the injured area by enhancing cell migration and expansion ability [36]. These results are consistent with our findings. In CCK8 and colony formation assays, we further verified that PRP at the density of 100 × 10^6^ platelets/ml accelerated cell proliferation. The results of scratch and transwell assays also confirmed that cell migration and wound healing were markedly enhanced after cocultured with PRP, but the PPP group displayed no obvious effect on cell proliferation and cell migration. PI3K/AKT signaling is a classical pathway for cell proliferation and survival, and there is evidence that it is activated by HSPs [37]. Upregulation of p-AKT is likely another important component of MSC therapy, since it is known to be involved in the regeneration and repair processes of damaged kidney tissue [38]. Immunofluorescence and western blot were used to measure the level of related proteins, and our data showed that PRP could attenuate the level of GSK-3*β* and enhance the level of *β*-catenin by activating the PI3K/AKT signaling pathway in BM-MSCs. The protein level of N-cadherin, Vimentin, *α*-SMA increased, while the protein level of E-cadherin was reduced. When we added the AKT inhibitor LY294002, the expressions of E-cadherin, N-cadherin, Vimentin, *α*-SMA, and AKT were completely blocked and cell migratory behavior significantly decreased. We were unable to detect the expression of AKT and GSK-3*β* phosphorylated protein, which is a major limitation of this study. Thus, we only speculated that the activation of PI3K/AKT signaling may be the mechanisms by which PRP promotes the migration of BM-MSCs. Furthermore, our studies showing that BM-MSCs pretreated with PRP have a greater ability to proliferate and migrate.

In summary, our findings have clearly demonstrated that low concentrations of PRP can stimulate proliferation and migration of BM-MSCs in vitro. Our study clearly indicates the potential of PRP for clinical treatment, but the clinical application of PRP needs more experimental support.

## Data Availability

The data used to support the findings of this study are available from the corresponding authors upon request.
